# Utilization of the Validated Windshield Material Model in Simulation of Tram to Pedestrian Collision

**DOI:** 10.3390/ma14020265

**Published:** 2021-01-07

**Authors:** Stanislav Špirk, Jan Špička, Jan Vychytil, Michal Křížek, Adam Stehlík

**Affiliations:** 1 Regional Technological Institute, University of West Bohemia in Pilsen, Univerzitni 8, 306 14 Pilsen, Czech Republic; spicka@ntc.zcu.cz (J.Š.); krizek4@rti.zcu.cz (M.K.); stehlika@rti.zcu.cz (A.S.); 2 New Technologies—Research Centre, University of West Bohemia in Pilsen, Univerzitni 8, 301 00 Pilsen, Czech Republic; jvychyti@ntc.zcu.cz

**Keywords:** tram, pedestrian, crash, windshield model, HIC

## Abstract

The rail industry has been significantly affected by the passive safety technology in the last few years. The tram front-end design must fulfill the new requirements for pedestrian passive safety performance in the near future. The requirements are connected with a newly prepared technical guide “Tramway front end design” prepared by Technical Agency for ropeways and Guided Transport Systems. This paper describes research connected with new tram front-end design safe for pedestrians. The brief description of collision scenario and used human-body model “Virthuman” is provided. The numerical simulations (from field of passive safety) are supported by experiments. The interesting part is the numerical model of the tram windshield experimentally validated here. The results of simulations are discussed at the end of paper.

## 1. Introduction

**Figure 1:**
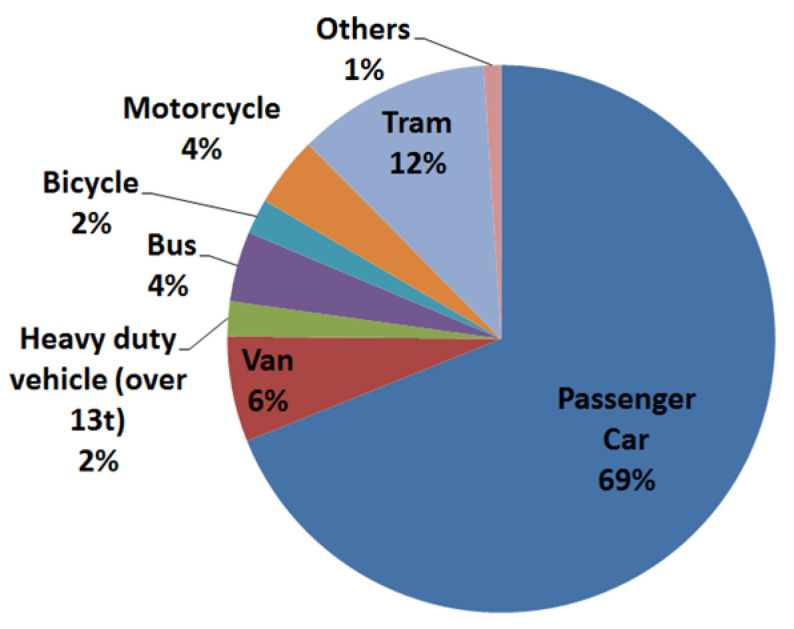
Distribution of the vehicle–pedestrian accidents in the Czech Republic in 2009–2014 [6].

This paper is an extended research paper of the conference presentation EAN 2020 [1] prepared for the special issue “Selected Papers from Experimental Stress Analysis 2020” in Materials journal. Traffic injuries represent one the most significant cause of the death around the world [2]. Moreover, pedestrians are the most vulnerable road users and they are exposed to a high risk in the collisions with the vehicles. The statistical data show that pedestrians are still responsible for the second biggest number of fatalities and injuries on the road: about 2000 in the Czech Republic [3,4], 40,000 in the EU, and 1.25 million around the globe annually [2,3,4,5,6]. The recent studies in Europe indicate that the passenger cars are one of the most often involved in the collisions with the pedestrians. Figure 1 summarizes the distribution of the vehicle type participating in the pedestrian collisions in the Czech Republic for the years 2009 to 2014 [7]. However, the number of pedestrians involved in tram crashes is not insignificant (about 12% of total cases in CR between 2009 and 2014).

The main aim of this paper is the utilization of the validated numerical model of human in the pedestrian–tram collision scenario. There is a new technical guide “Tramway front end design” currently under preparation by Technical Agency for ropeways and Guided Transport Systems [8]. This document is focused also on the guidance of the legislation of the new tram vehicles, with respect of the pedestrian safety and tram front-end design crashworthiness. The main aim is a condition of survivability of the pedestrian when the frontal crash occurs. The conditions of the collision are defined, as well as limits and threshold of the mechanical quantities describing the injury risk of the human. The legislative document is based on the automotive industry and its EuroNCAP regulation [9,10]. As the head is the more likely to be seriously injured in the collision with the tram, the main interest of the legislation is paid to its injury, monitored with the so-called Head Injury Criterion (HIC), where the peak of linear acceleration of the head center of gravity COG is to be the most responsible for the injury [11]. From the experience with tram development and from the experience with the automotive research, the authors assume that one of the most important part of the of the tram front end responsible for the level of head injury is the windshield [12,13,14]. This work is tightly connected with the research project focused on the development of the new tram, in the cooperation with worldwide tram developer acting in Czech Republic. As the design and the material need to be optimized, the mechanical behavior of all materials is required to build a suitable numerical model that will be used during the developing process. The main attention here is paid to windshield of tram as significant safety feature. The windshield consists of three layers (glass, PVB foil, and glass), and these must be described with the appropriate numerical model. The validated layered glass model from the automotive industry is used here and modify for the tram. In order to test its fidelity, the experimental test was done and compared with its numerical model. 

The experimental test here is the pendulum test, where the rigid steel ball impacts the tram glass. The mass, diameter, and velocity of the impactor was defined in order to be close enough (similar kinetic energy) as a human head during the frontal collision with tram. The material specification, complete description of the test and level of the fidelity is described later. 

The model of the windshield is then utilized in the full model of the tram (its front part) and together with the numerical model of the human Virthuman, the collision scenarios can be simulated. The Virthuman model is a model of the human body validated under specified conditions [15,16,17,18,19], and it has been successfully used in automotive research for the specified collision scenarios in correspondence with the tram regulation [8], to help in the process of the development of the new tram design. As this regulation defines the scenario, in which, the new vehicle is tested, the aim was to prepare and test this model, whether is a suitable tool and can help in the development of the new and pedestrian safety tram. 

This paper is firstly focused on the material model of the windshield suitable for a Visual Performance Solution (VPS) [20]. There is an assumption, that the layered glass of a windshield is equivalent to the passenger vehicles, only the thickness of the glass and PVB foil has changed. Thus, the authors are using validated automobile windscreen model, modified it in order to represent a tram one, and if necessary, some material parameters optimization will be performed, to get the correct material models. In order to verify the numerical simulation, the experimental pendulum drop tests were done and results (especially crack propagation is being tested). 

Second part of the paper uses the acquired windshield model in the full FE model of the tram required for the testing of the safety feature [8], where the pedestrian to vehicles collisions are defined. The aim is to prepare, to test and to verify this numerical model to be useful tool in the process of development of the tram front end design. The testing conditions as well as limits and threshold for the outputs are discussed. The new design and material of the tram, developed within the research group consisting of the research institute, tram producer, and testing institutions, is utilized here, and its description is provided here. Finally, the results of the tram vehicle, consisting of the validated windshield model, Virhuman model in the defined configuration [8] are presented. 

The tram regulation defines the mechanical quantities on a human and its limits, to rate the safety of the vehicle, in case of collision with the pedestrian. In case of the human head injury, the regulation concerns only of the HIC criterion and the max threshold is 1000. For such purpose, the appropriate model of a human must be used, to get required results. The human model must have adequate level of biofidelity, must be validated for such purposes and must have the appropriate outputs. One of the suitable human model is a hybrid approach model Virthuman, developed for universal use with validation based on real human body behavior. Moreover, the authors have a great experience with the development and utilization of this model. The Virthuman model is briefly described in the next section and then, used in the defined crash scenarios. The list of the human body models can be found for instance in [21]. 

## 2. Material Model Description

The laminated glass generally composed of two outer layers of glass and one inner layer of polyvinyl butyral (PVB) [22,23]. The windshield modeling method here is based on the studies connected with the automotive industry, where the windshield has been studied and tested, and its material model was developed. The authors here defined an assumption: that the tram and passenger windshield are made of the aforementioned materials and only the thickness varies. Thus, the model from the automotive industry is further used as an initial material model for a tram modeling. The passenger car windshield is used here and further modified to better describe behavior of the tram windshield in the experiment done here. The laminated layered glass FEM is modeled as three layers of shell elements connected by tied contact (rigid node-segment link). The PVB foil is modeled as an isotropic nonlinear viscoelastic shell element of Maxwell type:

(1) $$\sigma =k\left(1-e^{-w\varepsilon }\right)\left(1+h_{1}\varepsilon +h_{2}\varepsilon^{2}\right){\left(\frac{\dot{\varepsilon }}{{\dot{\varepsilon }}_{ref}}\right)}^{m}$$

where $\dot{\varepsilon }$ is the plastic strain rate; ${\dot{\varepsilon }}_{ref}\;$ is the reference strain rate; and *k, w, m, h*_1_*,* and *h*_2_ are material constants. The glass is modeled as linear elastic material with a brittle failure criterion. For the fracture definition, the Rankine criterion is used [24]: fracture occurs when the maximum principal stress exceeds the critical value,

(2) $$\sigma =\left[\begin{array}{ccc} \;\left(1-d_{1}\right)\sigma_{11} & \left(1-d_{max}\right)\sigma_{12} & \left(1-d_{max}\right)\sigma_{13}\\ \left(1-d_{max}\right)\sigma_{12} & \;\left(1-d_{2}\right)\sigma_{22} & \left(1-d_{max}\right)\sigma_{23}\\ \left(1-d_{max}\right)\sigma_{13} & \left(1-d_{max}\right)\sigma_{23} & 0 \end{array}\right]$$

where *σ* is the damaged stress tensor, *σ*_11_*,σ*_12_*,..,σ*_23_ are components of undamaged tensor, *d*_1_ and *d*_2_ are damage values in two directions, and *d_max_* is maximum of *d*_1_ and *d*_2_.

## 3. Experimental Testing and Validation of Windshield Numerical Model

The main idea here was to create an experimental setup that can be used for the validation and verification of the windshield model for the purposes of this work (i.e., impact of the human head, with the travelling speed of the tram). The standard process of head testing used the normalized head impactor [25,26], head FE model [27], or dummy model [28]. However, for our purposes, only the similar impact conditions (impactor stiffness, mass, velocity, and energy) must be satisfied, as the equivalent numerical model are created to compared with experimental results. The experimental testing with the safety laminated glass has its limitation. It is not possible to get tensile sample from producer (final glass cannot be cut), the brittle glass is not suitable for tensile test, PVB foil separated from glass has disrupted surface and the adhesion between glass and PVB foil is not known and cannot be experimentally tested (PVB material adheres to glass through hydrogen bridges [29]). In order to make the model simpler and to decrease the calculation time, the 2D elements are used here. This simplification has its basement also in automotive, aviation or civil engineering research, and thus can be used also here and should not significantly influence the final results [30,31,32,33,34]. 

**Figure 2:**
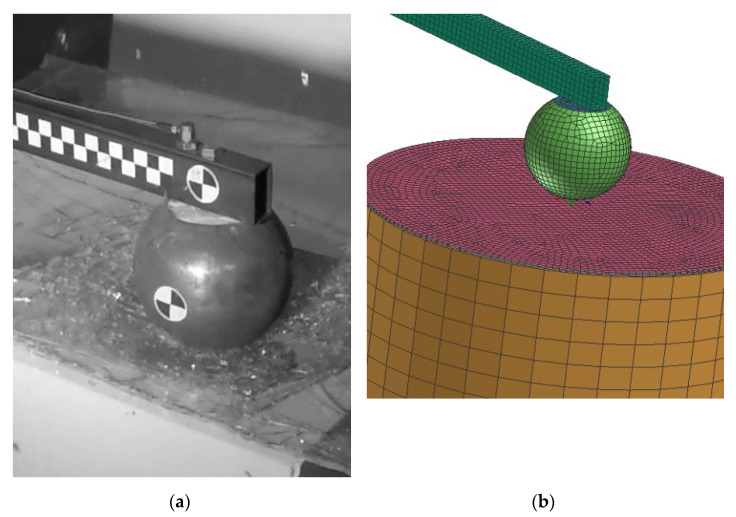
Experimental test of windshield (**a**) and its numerical model (**b**).

In order to validate the material model for this purpose, the simple pendulum test and its numerical model are built, see Figure 2. The pendulum is made of steel S235 profile (50 × 30 mm^2^, thickness 2 mm, length 2000 mm and 4.41 kg of mass) and the ball impactor (150 mm of diameter and 4.7 kg of mass). The circular section of the tram windshield is placed on the extruded polystyrene (with known properties) with diameter 300 mm. The steel impactor falls on the sample of the glass from the height of 2000 mm. The tested glass was a rea tram-layered glass (3 mm glass, 0.9 mm PVB foil, 3 mm glass). More than 10 tests were performed to get statistically significant results. In most of the tests, the ball was falling from full height (2000 mm). The friction between impactor and glass was 0.5 and the glass model was constrained to polystyrene basis via tied constraint (rigid node to node). The impactor in the model was constrained with revolute joint to the basic, and loaded with the initial velocity to COG. In order to speed up the calculation, the free fall of the pendulum was neglected, and the simulation starts when the impactor is just about to hit the glass. The initial velocity was calculated and verify with the data from experiment.

**Figure 3:**
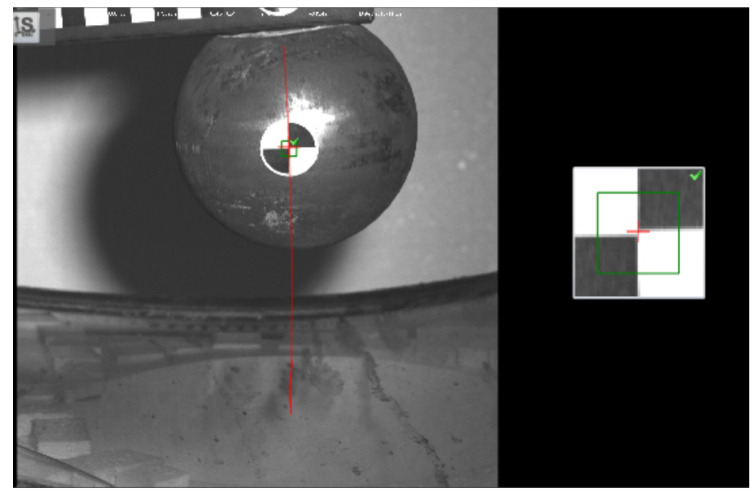
The impactor path reconstruction from target focusing.

Some tests were executed from a smaller height and also with initial crack on glass or with different shape and size of glass. These experimental results are not described here. However, the example of the experimental curves is presented in the Appendix A. The condition of smaller initial height allows us to validate the model also for range of initial velocities. The windshield model was validated only for full height with the impact velocity 6 m/s. The acceleration was recorded with Brüel & Kjær accelerometer (4533-B 10 mV/g, ±500 g) (Nærum, Denmark). Displacement of the impactor was recorded with high-speed camera (Photron fastcam SA X2 RV) (Tokyo, Japan) and reconstructed with the target focused method. In each photograph, the target (Figure 3) on the rigid impactor was selected and monitored. If the size of each pixel is known, than the displacement (and also its derivatives) can be calculated.

**Figure 4:**
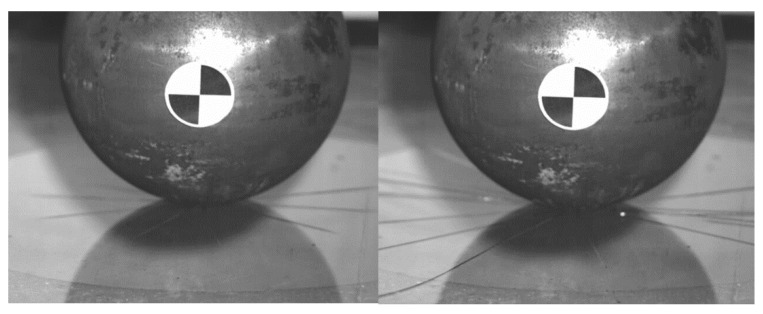
The radial crack propagation after the first contact of impactor.

**Figure 5:**
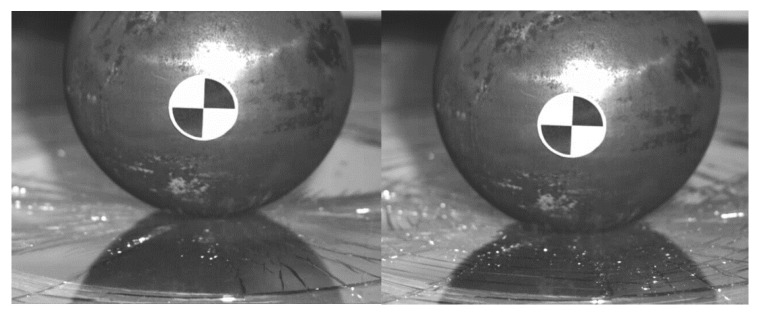
The concentric crack in the second state.

**Figure 6:**
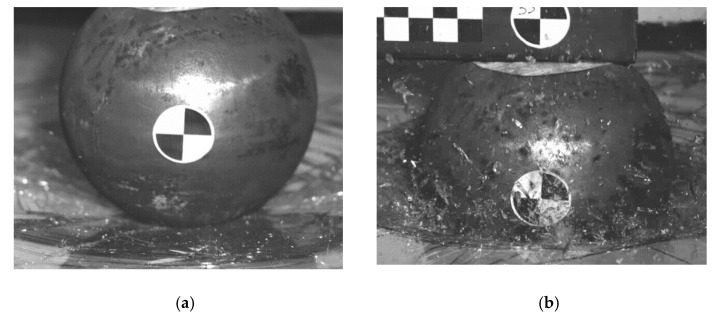
The ultimate glass fracture (**a**); maximum penetration (**b**).

**Figure 7:**
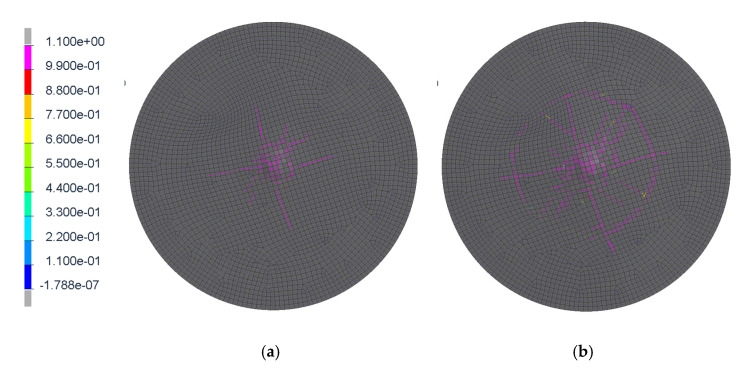

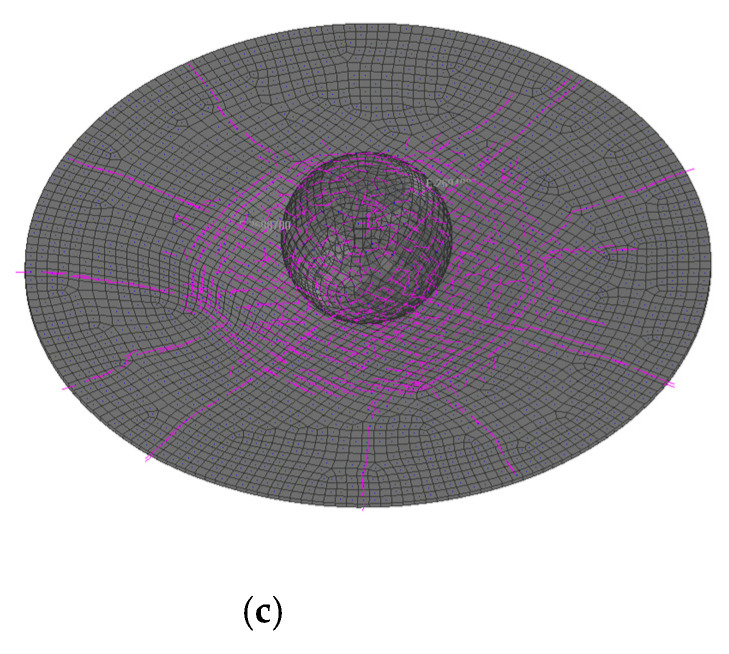
The damage tensor directions; first radial cracks (**a**); secondary concentric crack (**b**); final ultimate fracture during the maximum impactor penetration (**c**).

This kinematics conditions were set up to be similar with pedestrian head impact during collision with tram (initial speed 20 km/h). The impactor is considered as a rigid body, as its purpose is to validate the glass model (not to evaluate head injury prediction). It is clear that this model cannot exactly predict the crack shape in detail, but it has been discovered that in repeated experiments and simulations the influence of crack shape difference is insignificant. In these phenomena, the crack shape has some general similarities in radial and circular direction, see Figure 4, Figure 5, Figure 6 and Figure 7. Moreover, the acceleration results are influenced by the steel rod oscillations. This is one of the possible improvements for the further experiments. The glass rupture propagation is visible on the figures below.

**Figure 8:**
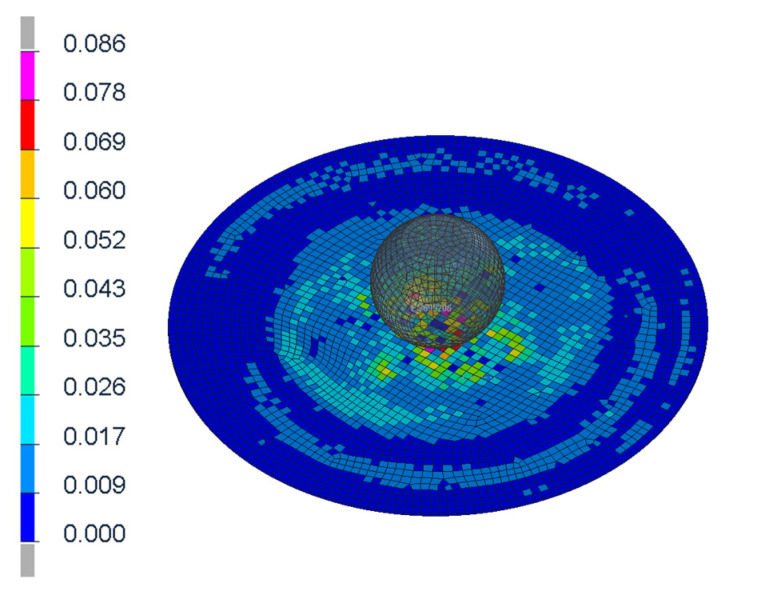
Discribution of Von Mieses stress of the glass after the first contact.

**Figure 9:**
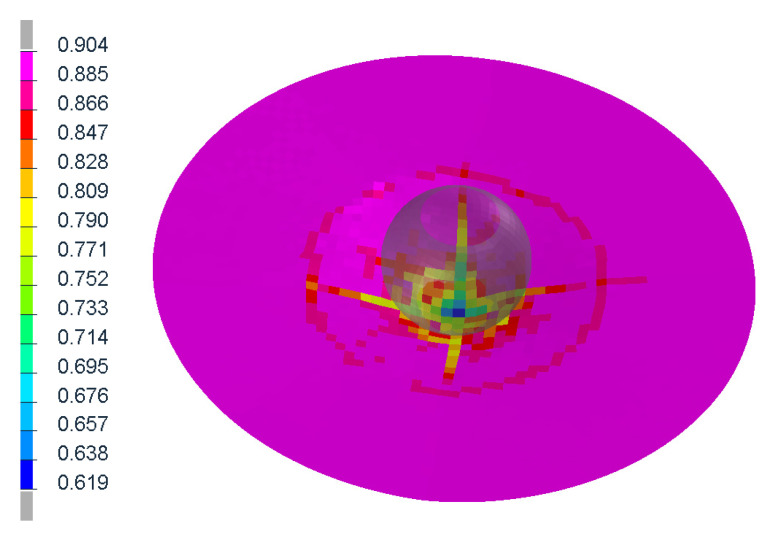
Thickness of the PVB foil at the maximum of penetration.

The figures above (Figure 4 and Figure 6) show the visible the rapture mechanism, crack propagation of the glass and the ultimate rupture during the maximum penetration. Similar behaviors were observed also in the simulation (Figure 7). Stress distribution and thickness of the PVB foil are shown in the Figure 8 and Figure 9, respectively.

As was described above, the model of the passenger car windshield was used here as an initial guess. The thickness of the layers was modified based on the real tram data in order validate the model. First set simulations confirmed this theorem, as the simulated results were very close to the experimental ones. Thus, there were no requirements of numerical optimization (tuning of the material model parameters) to get the proper level of fidelity. Only minor modifications were done on the windshield model.

**Figure 10:**
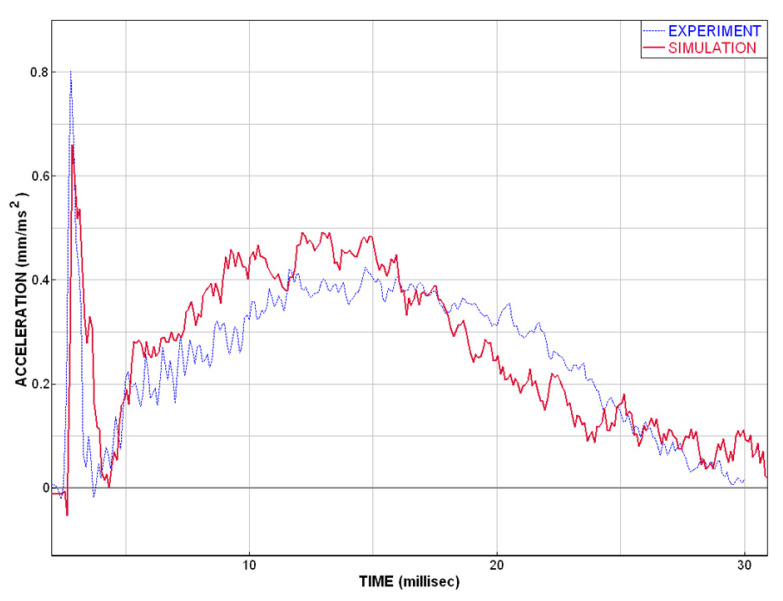
Plot of the acceleration vs. time.

**Figure 11:**
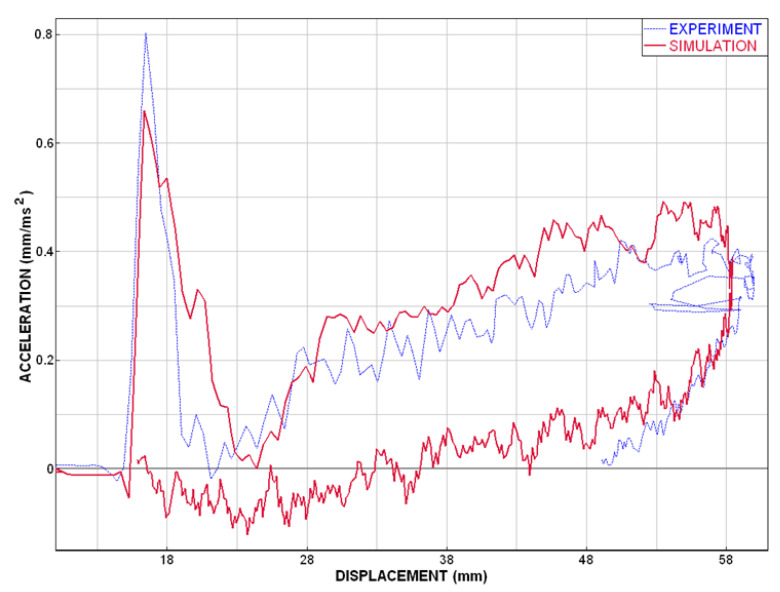
Plot of the acceleration vs. displacement.

The comparison of the experimental data and numerical model (described above) shows a good correspondence. This coincidence is adequate and good enough for the purpose of safety simulations and for head impact injury predictions in case of windshield–head impact. The acceleration–time curves are very close to each other; see Figure 10, which indicates also similar result of HIC criterion. The deformation characteristics result in a good agreement of experimental and simulation curves Figure 11.

## 4. Parameters of Validated Material Model

The description of material models of the glass and PVB foil, respectively, are presented below (units: mm kg ms), see Table 1 and Table 2. These material parameters are used for tram windshield in simulation with three layers (3 mm outside glass, 0.9 mm PVB foil, 3 mm inside glass) connected with tied links. The presented material parameters are for the VPS software, where the entire model is built. The VPS software includes the Glass model, designed exactly for a modeling of the glass. The PVB foil is defined as a linear viscoelastic 2D material.

## 5. Collision Scenario

The collision scenario defined in the technical guide is based on the statistical data of the tram to pedestrian collisions and also follows the automotive safety scenarios, defined in EuroNCAP [9,10]. The collision scenario is defined as a moving tram hitting the pedestrian (moving or standing) from his side. The pedestrian is moving perpendicular to the tram trajectory, in front of its front end. The technical report divides the impact into three phases, where the first phase is considered as an impact of the vehicle to the pedestrian. Second phase is an impact of the pedestrian onto the ground and the third impact phase deals with the scenario, where the pedestrian lays on the railway (ground) and can be overrun with the vehicle. The scope of the technical report is focused on the first and third phases. The second phase is connected mainly with urban and civil engineering and material of the surroundings (grass, concrete, pavement, asphalt, etc.) and thus it is not examined here.

- First collision scenario (type A): the pedestrians involved in the collision are specified to be mid-size male (175 cm, 78 kg—50th percentile) and 6 years old (YO) child (110 cm, 24 kg). The report also defines possible impact area and impact zones, with respect to the shape of the vehicle, for more specification, see the new regulation [8]. The collision scenario evaluating the first impact consider the tram moving with the initial velocity equals to 20 km/h and pedestrian standing still, left side to the vehicle, one step forward (not specified which leg to be forward) and the lateral position of the pedestrian relative to the vehicle has two specifications (H-point with respect to the tram):
  ○ 15% value of half of the tram width
  ○ 50% value of half of the tram width

The vehicle does not stop (not loaded with any deceleration pulse) only energy lost due to the impact. The pedestrian injury risk is monitored only with the Head Injury Criteria (HIC) [10], which should not exceed threshold of 1000. The second impact (pedestrian to the ground) is not included here, as it is connecter more with the urban engineering than traffic engineering.

- Second collision scenario (type B): (overrun of the pedestrian) is tested via four scenarios (each of them with the adult and child dummy). For this particular test, the dummies are specified to be the adult rescue dummy” (183 cm, 75 kg) and the child rescue dummy” (122 cm, 17 kg). The testing scenarios are defined as follows.
  ○ Test 1: transverse to the rail, centered
  ○ Test 2: transverse to the rail, off center (hip on the rail)
  ○ Test 3: lengthwise on the rail, centered (feet pointing towards the tram)
  ○ Test 4: lengthwise on the rail, off center (hip on the rail, feet pointing towards the tram)

This technical report also describes the protective technology to be used and how to be used, the distance between dummy and vehicle during test etc. The initial velocity of the tram in this collision scenario is 25 km/h and after reaching specified position, it starts to break within emergency breaking until it stops. The objective of this test is to verify capabilities of the vehicle during crash, with the following parameters.

○ To stop any part of the rescue mannequin before the first wheel set
○ Not to jam the rescue mannequin at its thighs, chest, or head
○ Not to sever one of the rescue mannequin’s limbs so that the rescue mannequin should remain intact
○ To push the rescue mannequin away so that it does not come into contact with the wheels
○ Not to trigger any debris or fracture on impact with the rescue mannequin (risk of aggravating injuries)

The full test protocol is available in the technical report, where all settings of the test conditions are specified. The conclusion of the test indicates whether it meets the objective or not. There is no threshold value specified to pass the tests. Only the position of the pedestrian with respect to the tram is monitored.

## 6. Human Body Model Virthuman

**Figure 12:**
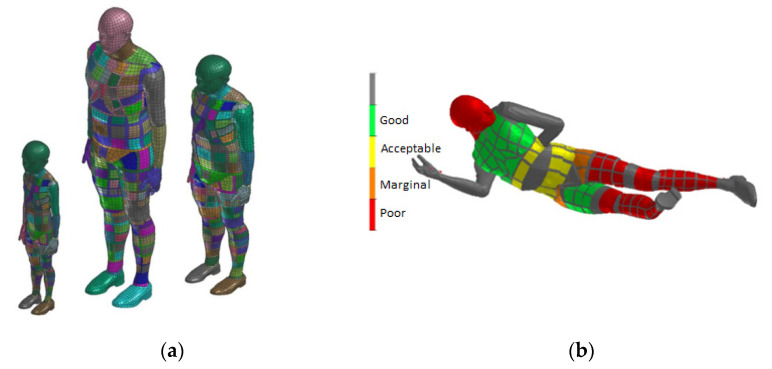
Virthuman model: Scaled model. 6-year-old child, 110 cm, 17 kg; 40-year-old male, 190 cm, 100 kg; 25-years woman, 50 kg (**a**); Injury risk rating (**b**).

In order to represent a pedestrian in the collision scenario, the Virthuman model is considered. It is a virtual human body model where the skeleton is built based on the multi-body structure (MBS). The outer surface of the model consists of deformable segments that are connected to the skeleton via nonlinear springs and dampers to account for deformability of soft tissues. Individual rigid bodies of the MBS structure are interconnected via kinematics joints. Moreover, additional “breakable” joints are considered in lower extremities to include the possible fractures of both femur and tibia of the pedestrian in the collision scenario. The model has been validated extensively to ensure its boofidelity in the particular scenarios, connected mainly with the automotive industry [15,16,17,18,19]. The basic reference model (50th percentile male) can be scaled using the parameters of height, weight, age and gender. In this case, the 50th percentile male was used corresponding to the Hybrid III dummy (male, 172 cm, 78 kg). The scaling algorithm has its basement in the wide database of population in Czechoslovakia in the 1980s, where anthropometric dimension of more than 10,000 people were measured [35]. Thanks to the MBS structure of the model, the model is easy to position in any desired position respecting real human anatomy and physiology (range of motion of the real joint [36]). In this case, the positions defined in the chapter “Collision scenario” were considered for the crash scenario. Virthuman model includes an embedded algorithm in the model to evaluate standard injury criteria for individual body parts as defined by EuroNCAP testing procedures [10]. During the post processing, the particular injury criteria is being checked and the individual body segments are colored based the threshold of such criterion (Figure 12). In case of the head, the Head Injury Criterion is used in this study to predict injury sustained by the pedestrian in the collision with the tram [11]. 

## 7. Numerical Model of Collision with Windshield Material Model

For the dynamic structural analysis (with significant nonlinearities), the explicit integration method is used. The tram to pedestrian collision is modeled with numerical software Visual Performance Solution (VPS) [20] and human body model Virthuman described above is used. The model of the vehicle is created mainly with quad and brick elements with one gauss integration point. The model was discretized into 1.6 million elements with the smallest element characteristic length 2 mm (leading to a time step 5 × 10^-6^ ms). The contacts and links (node to segment connection) are realized by penalty algorithm. The simulation time of the defined scenario is 390 ms. The mechanical properties of steel S235 [37] and steel 1.4301 are known. The top shell cover is made from polymer (acrylonitrile butadiene styrene) with acceptable fire protection and recycling possibilities. Unfortunately, mechanical properties of this material used in simulation are confidential (courtesy of the company) and cannot be provided. The Virhuman model is loaded only with gravity, while the vehicle is loaded with the specified velocity (constant during entire simulation—type A; constant until specified time—type B). Contact between the human and the tram is defined as an asymmetric node-to-segment contact with the friction coefficient equals to 0.3. The connection between the structural compartment of the vehicle is realized with the rigid (tied, rigid spot-weld, rigid constraint) or deformable (glued) connection. For instance, the glass is connected to the frame by glue, modeled as deformable solid, connected to frame.

There are two different setups of the simulations: The first type is a collision with standing pedestrian. The second one is a collision with pedestrian lying on the ground. All simulations are performed with the same human and tram model, respectively. In previous section, the windshield model was tested with the experimental set up, to proof its fidelity for this particular scenario. The main aim here is to apply the simulations from the field to provide passive safety to the pedestrian. The crashworthiness of the newly developed tram design is evaluated here, with respect to defined regulation for tram safety [8]. The structure of the Virthuman model is based on hybrid approach (MBS basic skeleton and rigid super elements connected via springs and dampers to the basic structures) to account for the local deformations. Consequently, the particular body segments do not catch the full local deformation, such as full FE model (GHBMC or THUMS [21,38]). However, the deformation is represented with the deformation of the spring and dampers, and the motion (displacement, velocity, acceleration) of the COG is close to real motion (validated model). Thus, the user cannot see the deformation via visual observation, but the acceleration curves and HIC value are adequate. 

## 8. Results

**Figure 13:**
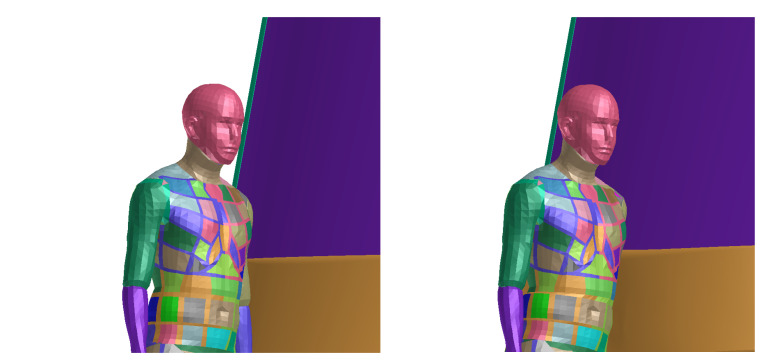
Results of simulation (in time 0 and 25 ms) where the head impacts the windshield.

**Figure 14:**
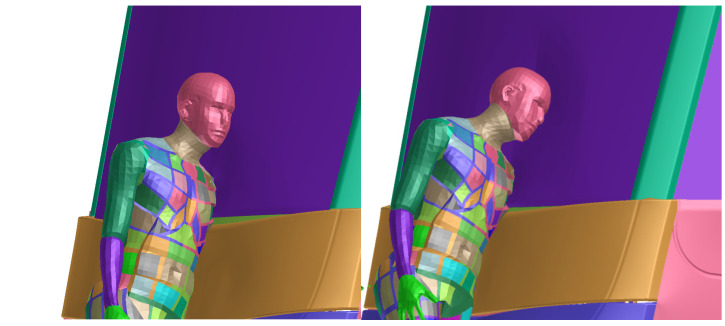
Results of simulation (in time 50 and 75 ms) where the head impacts the windshield.

**Figure 15:**
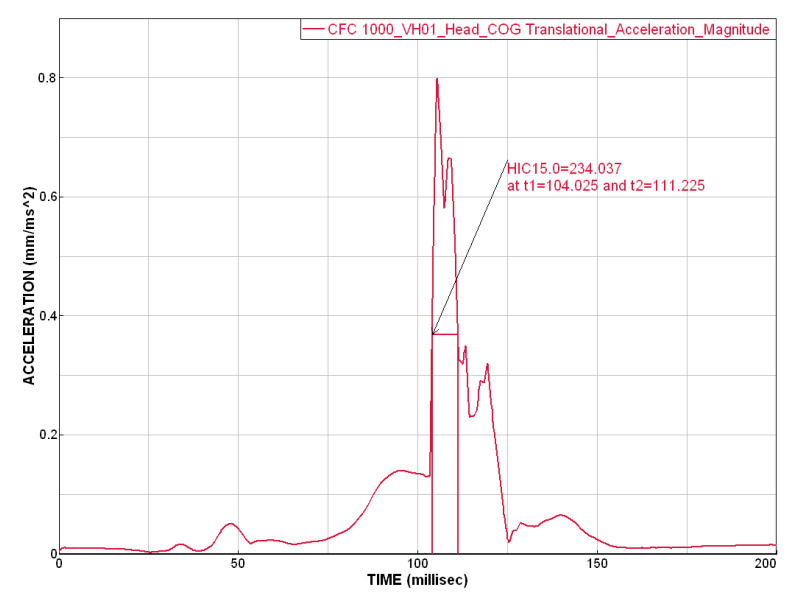
Acceleration of the COG (center of gravity) filtered by CFC1000 filter with HIC15 injury criterion assessment.

**Figure A4:**
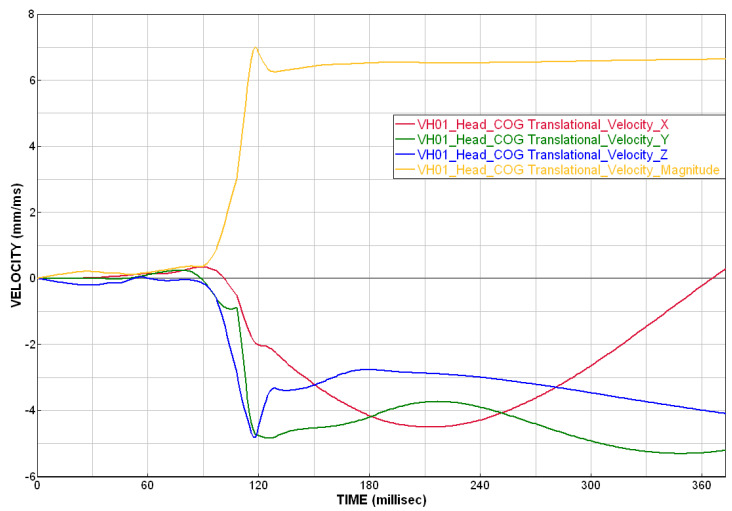
Velocity of the head in the simulation of collision scenario A.

The sequence Figure 13 and Figure 14 in the time shows the detail of the head impact to the tram front-end. This part of collision is the most important, as the head injury connected with significant severity occurs here. It is clearly visible that the head impact occurs directly to the windshield. However, the head acceleration does not exceed limit (Figure 15) of 0.8 m/s^2^ and the HIC criterion is only 234 (below the threshold of 1000). This indicates low or acceptable injury risk of the head. The peak in the curve is results from the rapid change in acceleration and velocity (in accordance with the crash impact theory: large magnitude in a short time). The velocity of the head during the impact does not have any threshold value (no specified criterion or limit); however, this value can help quantified the injury risk. In this particular scenario, the velocity of the head COG was about 7 m/s in magnitude, see Figure A4. 

**Figure 16:**
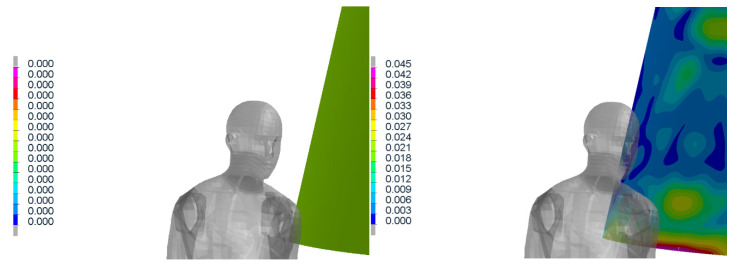
Results of simulation (in time 0, 40 ms) where the stress distribution of the glass is shown.

**Figure 17:**
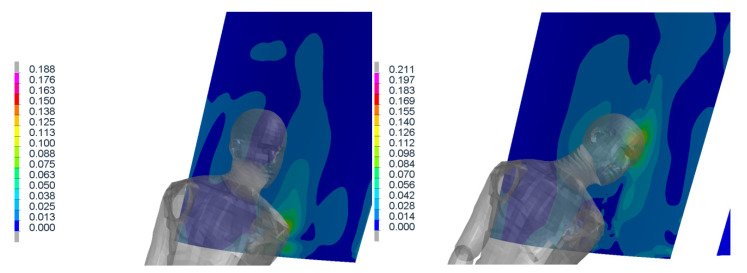
Results of simulation (in time 80, 120 ms) where the stress distribution of the glass is shown.

Figure 16 and Figure 17 show the stress distribution of the layered glass under the impact of the body (shoulder and head, respectively). 

The presented results of the pedestrian–tram collision are only an example of the calculated results. The aim of this paper was to validate the windshield model for this scenario, and to use this model in the full model of the tram and pedestrian collision. Moreover, this work is tightly connected with the research project developing a brand new tram (in cooperation with the worldwide producer) [14] and is based on the safety requirement [8]. Thus, only the crash configuration defined in this regulation is analyzed. However, from the authors’ experience, we can expect a different dynamic an injury risk of the pedestrian for different initial condition (lateral position of the pedestrian with respect to the vehicle or rotation of the body) [13]. Moreover, the location of the first contact can significantly change the results (effect of the shoulder and arm or elbow respectively). All these aspects are considered in the tram design development, however not presented here. The paper here is testing the crashworthiness of the new vehicle in the conditions specified in the European regulation [8]. 

**Figure 18:**
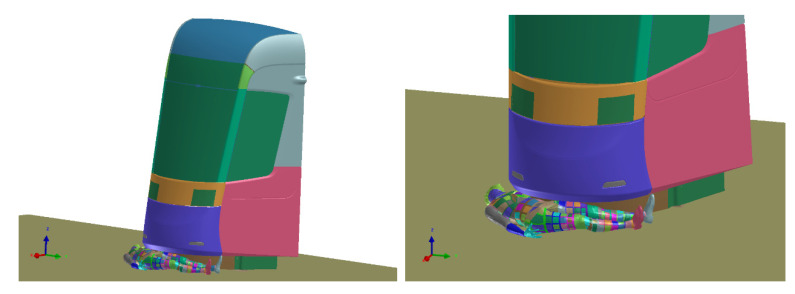
Crash scenario B, overriding with adult rescue dummy, transverse to the rail, centered.

**Figure 19:**
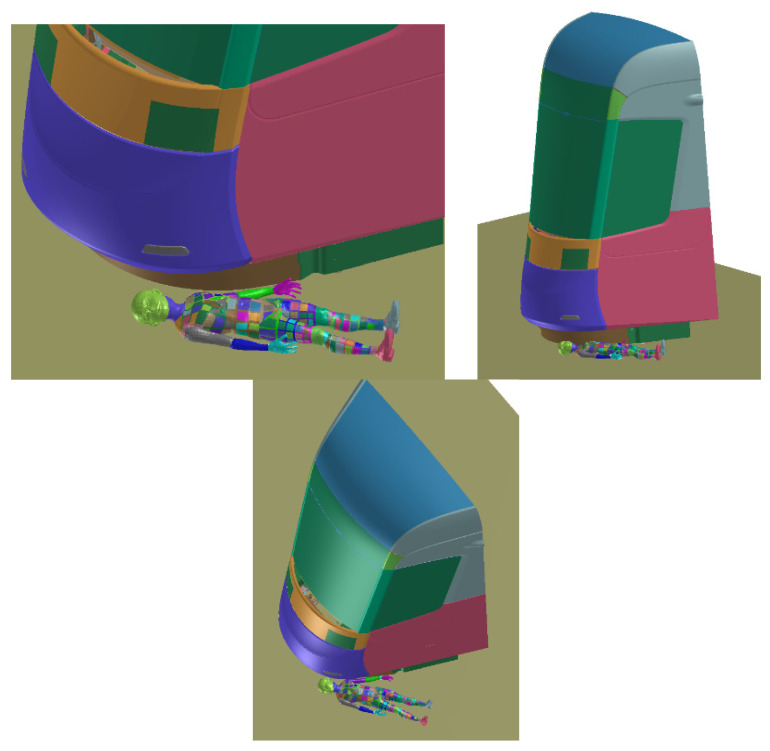
Crash scenario B, overriding with child rescue dummy, transverse to the rail, off-centered.

The collision of tram and laying pedestrian shows how the requirements for the pedestrian anti-crush mechanism are met (Figure 18). With the advantages of the simulations, the effect of mechanism with correct clearance is visible. It can be said that the injury of laying pedestrian during collision with tram without pedestrian anti-crush mechanism are crucial (the most of trams has no pedestrian anti-crush mechanism see Figure 19). The design of a new tram can save many lives and significantly reduce number and severity of injuries. Based on the calculated simulations, the new tram is passing the requirements.

## 9. Conclusions

This paper contains a description of experimental material testing and its validation for the purpose of pedestrian collision. As the numerical model of the passenger car is available, the assumption of its similarity with the tram glass was defined. We assume that the materials are equivalent and only the thickness of the particular layers (inner and outer glass and PVB foil) varies. The experimental test of the sphere impact to the tram glass was performed and the results were utilized in the material testing and verification. Since only the minor modification of the car windshield was necessary, this statement was confirmed and the obtained model of the glass can be further used in the full tram model, for the scenarios of pedestrian impact. Furthermore, the impact of the head and its dynamic was the main concern, and thus, this head-like impactor test was appropriate. If the glass model would be used in other impact conditions (especially under different impact condition: change in the mass, shape or velocity of the impactor) a new validation would be necessary. The final material model of the glass and PVB foil for the VPS software is presented (material parameters table). The best comparison of simulation and experiment (for the mode of impact loading) is in macroscopic characteristics (like deformation characteristic). The simple pendulum test has some inaccuracies (see above), which can be further improved. The validated material model shows a good coincidence with the experiment. 

The windshield model is further used in the tram model. The collision of tram to pedestrian is described in second half of paper, with the respect to the newly defined certification conditions. The collision scenario was simulated with the advantage of the virtual human body model “Virthuman”. The injury of the pedestrian head (HIC) is highly influenced by the windshield behavior and thus the windshield was the main concern of this paper. The simulation (of pedestrian collision with full-scale tram face) indicates that the defined tram to pedestrian crash scenario results in the HIC value (234) significantly smaller than the threshold limit of 1000. The Virthuman model is not a full FE model of the human body, and of course it has some limitations. However, in the case of traffic accident and human injury, especially injury of the head, it results in a good level of boofidelity. Thus, the results of the tram to pedestrian collision are reliable for this particular research. The first results of the experiments suggest very similar material behavior of tram and road vehicle windshield. Therefore, it is possible to use the material model of glass and PVB foil from an automotive industry with modified thickness and minor modification of the material parameters also in the tram model. The results of the simulation (with experimentally validated material) indicate that the windshield is feature with good crashworthiness. 

The new design of tram with low height of bottom windshield show the good behavior with respect to the pedestrian injury both in the frontal crash (scenario A) and overriding of the laying pedestrian (scenario B). However, the further research is still an ongoing task, and full vehicle testing must be done. 

## Figures and Tables

**Table 1:** Parameters of material model GLASS.

Mat Type	Density	Belyts.-Tsay Reduced Integration	Stiffness Elastic Hourglass	Quadratic Viscosity Multiply	
126	2.5 × 10^−6^	0	0	1	
E	NU	Membrane Hourglass	Out of Plane Hourglass	Rotation Hourglass	Trans Share
70	0.2	0.01	0.01	0.001	0.8333
Sigmac	Time Filter	Stiff Damping			
0.031	0.01	0.1			

**Table 2:** Parameters of material model PVB-foil (nonlinear viscoelastic shell model).

Mat Type	Density	Belyts.-Tsay Reduced Integration	Stiffness Elastic Hourglass	Quadratic Viscosity Multiply	
121	1 × 10^−6^	0	0	1	
E	NU	Membrane Hourglass	Out of Plane Hourglass	Rotation Hourglass	Trans Share
9	0.39	0.01	0.01	0.001	0.8333
G-Shell Param	K	M	H1	H2	W
	0.007	1.33	1.35	0	3

NOTE: The definition of all the defines parameters can be found in VPS manual [20]

**Figure A1:**
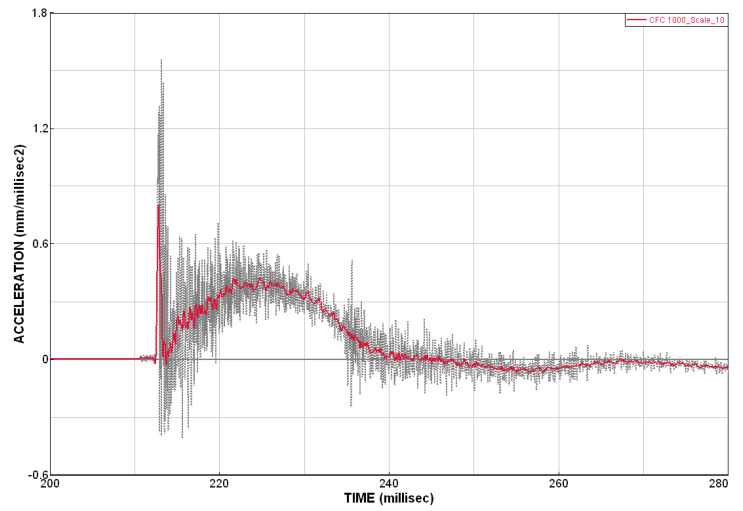
Original and filtered acceleration curve (sampling rate 48 kHz filtered with the CFC 1000 filter).

**Figure A2:**
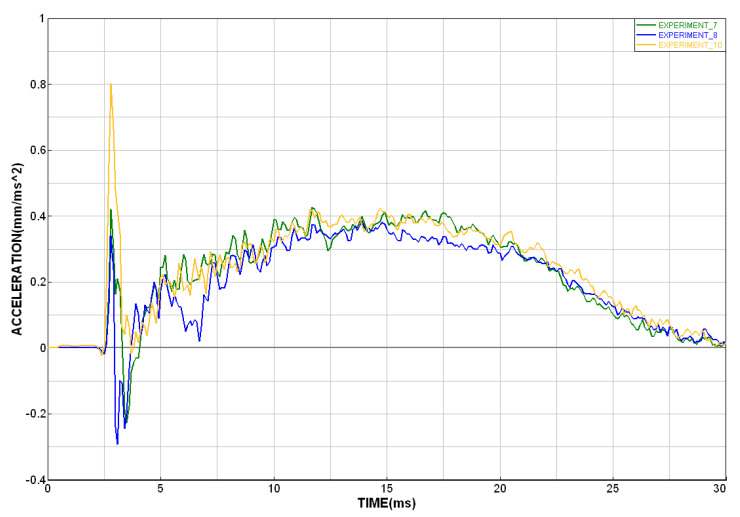
Examples of the experimental acceleration curves.

**Figure A3:**
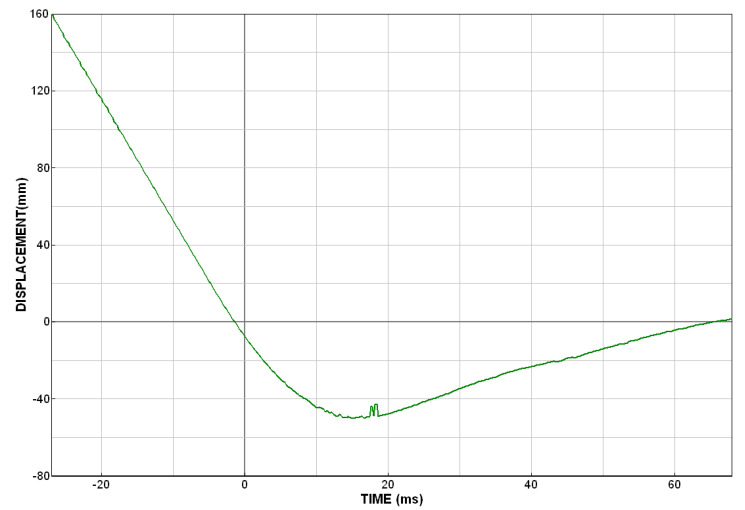
Displacement of the impactor COG, data from high-speed camera.

## Appendix A

The experimental results of the spherical metal impactor to the layered glass (windshield) are presenter here.
